# Isolated thrombocytopenia as the initial manifestation of childhood-onset systemic lupus erythematosus: A case report

**DOI:** 10.1097/MD.0000000000042874

**Published:** 2025-06-13

**Authors:** Fawzy M. Abunejma, Maaweya Jabareen, Wasef Alhroub, Bashar Qanaeer, Adam Zaro, Asrar Alhroub, Abrar Zaro

**Affiliations:** a Department of Pediatric Rheumatology, Faculty of Medicine, Hebron University, Hebron, Palestine; b Department of Pediatric Rheumatology, Faculty of Medicine, Alexandria University, Alexandria, Egypt.

**Keywords:** childhood-onset SLE, immune thrombocytopenia, pediatric autoimmune disorder, pediatric-onset SLE, systemic lupus erythematosus

## Abstract

**Rationale:**

Systemic lupus erythematosus (SLE) is a rare but serious autoimmune disorder that can present with diverse clinical manifestations, often making diagnosis challenging, particularly in pediatric patients. While thrombocytopenia is a known hematological manifestation of SLE, its occurrence as the sole initial symptom is uncommon.

**Patient Concerns:**

An 11-year-old male presented with a 2-week history of painless localized bleeding, ecchymosis, and petechiae. He also experienced mild knee joint pain but had no fever, weight loss, or other systemic symptoms. Despite initial treatment for immune thrombocytopenia (ITP), his platelet count remained persistently low, raising concern for an underlying autoimmune etiology.

**Diagnosis:**

After a thorough workup, including negative blood cultures and a bone marrow biopsy that revealed megakaryocytic thrombocytopenia, autoimmune testing revealed elevated antinuclear antibodies, antidouble-stranded DNA, and decreased complement C4 levels, leading to a diagnosis of childhood-onset SLE with isolated hematologic involvement.

**Intervention:**

The patient was initially treated with intravenous immunoglobulin for the management of ITP. Following the diagnosis of SLE, he was started on hydroxychloroquine, followed by azathioprine for immunosuppression. Inflammatory control was further achieved with prednisone therapy due to persistent hematologic symptoms.

**Outcome:**

The patient’s platelet count improved to 150,000/μL after 6 months of treatment. And the other laboratory was unremarkable, indicating effective management of SLE. The patient remained free of other organ involvement during follow-up.

**Lessons:**

This case emphasizes the importance of considering SLE in the differential diagnosis of pediatric patients with isolated ITP, especially when the clinical picture is atypical. Early autoimmune screening, including testing for antinuclear antibodies and double-stranded DNA, can facilitate early diagnosis and timely intervention.

## 1. Introduction

Systemic lupus erythematosus (SLE) is a chronic, multifaceted autoimmune disorder that can affect multiple organ systems, leading to a wide range of clinical manifestations. While SLE is most commonly diagnosed in adults, approximately 15% to 20% of cases occur in childhood, with onset before the age of 16.^[1]^ Childhood-onset SLE (cSLE) presents with diverse and often nonclassical symptoms, making early diagnosis challenging and frequently delayed. This delay in diagnosis increases the risk of long-term morbidity and mortality.^[2]^ Therefore, maintaining a high index of suspicion is critical, particularly in cases where clinical features deviate from the typical presentation.^[3]^

In rare instances, isolated immune thrombocytopenia (ITP) may be the initial manifestation of SLE. ITP is an autoimmune disorder characterized by the destruction of platelets due to autoantibodies targeting platelet surface proteins, resulting in platelet counts below 100 × 10^9^/L.^[4,5]^ While the association between ITP and SLE has been well documented, it is uncommon for ITP to be the sole presentation of SLE.^[6]^ In fact, the cumulative risk of developing SLE in children diagnosed with ITP is estimated to be 3.8% at 5 years and 6.5% at 10 years postdiagnosis.^[7]^ The median time from ITP onset to the eventual diagnosis of SLE is approximately 34.5 months.^[8]^ Given this, clinicians should consider the possibility of underlying SLE in pediatric patients with isolated ITP, and testing for markers such as antinuclear antibodies (ANA) and antidouble-stranded DNA (dsDNA) is essential for early detection.^[6]^

In our case, we present an 11-year-old male who initially presented with ITP and pancytopenia. Further investigation revealed positive SLE-related antibodies, leading to the diagnosis of cSLE. Notably, the patient exhibited no other organ involvement, and his presentation was limited to hematological manifestations. This case underscores the importance of considering SLE in the differential diagnosis of pediatric patients with isolated ITP.

## 2. Case presentation

An 11-year-old male patient presented to the pediatric clinic with a 2-week history of painless localized bleeding and the appearance of bluish patches across his body. The lesions, which initially presented as reddish, were localized to the upper and lower limbs as well as the chest. The patient also exhibited red sclera. The lesions were self-resolving. Additionally, the patient reported mild knee joint pain; however, there was no associated edema or other joint abnormalities. There was no history of fever, weight loss, excessive sweating, or bone pain. The patient had a positive family history of localized scleroderma, with a 6-year duration in a close relative.

**Figure 1.**
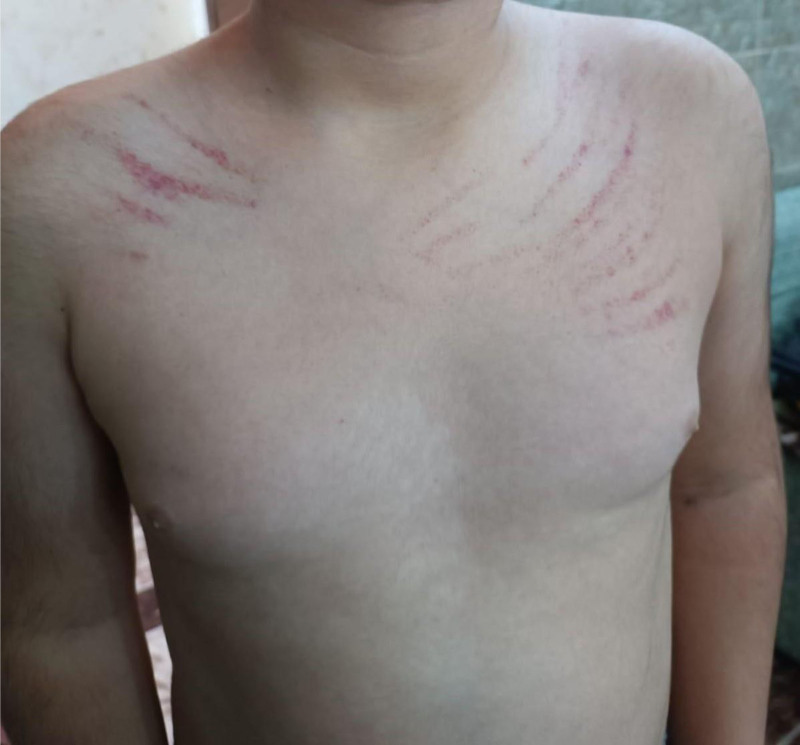
A photograph showing bilateral Petechiae, purpura, and ecchymosis on the upper chest.

On physical examination, the patient appeared weak and pale. Petechiae, purpura, and ecchymosis were noted on the chest (Fig. 1). A subconjunctival hemorrhage was also observed. The examination of the heart, lungs, abdomen, and ear, nose, and throat was unremarkable, and there were no signs of lymphadenopathy, organomegaly, or joint swelling.

Laboratory investigations, summarized in Table 1, revealed thrombocytopenia and a reduced white blood cell count. A positive direct Coombs test was noted, but blood cultures were negative for bacterial growth. Hepatic and renal function tests, lactate dehydrogenase, and coagulation markers were all within normal limits. Serological testing for HIV I/II, hepatitis B, and C was negative. A bone marrow biopsy revealed megakaryocytic thrombocytopenia.

**Table 1:** Laboratory investigations.

Parameters	Value	References range
Hgb	13.2 (↑)	11–13 g/dL
WBC’s	3400 (↓)	4000–11000/μL
Platelets	10000/μL (↓)	150000–400000/μL
MCV	82 fL (normal)	80–100 fL
Lymphocytes	1000 cells/mL (normal)	1000–4800 cells/mL
Direct Coombs test	Positive	
ENA profile	Negative	
ANA Titer IFA 1:80	Positive	
Anti-dsDNA	Positive (35.0 IU/mL) (↑)	>10 IU/mL
C3	97 mg/dL (normal)	90–180 mg/Dl
C4	4 mg/dL (↓)	10–40 mg/dL

ANA = antinuclear antibodies, C3 = complement C3, C4 = complement C4, dsDNA = double-stranded DNA, ENA = extractable nuclear antigen, MCV = mean corpuscular volume, WBC = white blood cell.

As the platelet count further decreased to 10,000/μL, the patient was readmitted and administered intravenous immunoglobulin at a dose of 0.8 g/kg. A transient increase in platelet count was observed, rising to 141,000/μL, but this improvement was short-lived, with a subsequent drop to 36,000/μL. Given the persistence of thrombocytopenia and the patient’s clinical manifestations, a workup for autoimmune causes was initiated. Testing for SLE revealed elevated levels of ANA and dsDNA, with decreased complement C4 and normal C3 levels, supporting the diagnosis of SLE.

After 2 months of treatment with 200 mg hydroxychloroquine daily, the patient was started on 50 mg azathioprine daily. During the 6-month follow-up, the patient’s laboratory results showed improvement: hemoglobin was 11.8 g/dL, white blood cell count was 3600/μL, neutrophil count was 4200/mL, lymphocyte count was 730/mL, platelet count was 150,000/μL, C3 was 87.2, and C4 was less than 4. Given these results, along with the persistence of the hematologic symptoms, the patient was started on 5 mg of prednisone daily. At this stage, a diagnosis of juvenile SLE with hematologic manifestations was confirmed without the involvement of other organ systems.

## 3. Discussion

SLE is a long-term autoimmune condition characterized by widespread inflammation that can impact multiple organs. It most commonly occurs in women, particularly those in their reproductive years.^[9]^ Where the clinical presentation ranges from mild, nonspecific symptoms to severe, organ-threatening complications. Critical manifestations of SLE can involve lupus nephritis, central nervous system impairment, and hematologic abnormalities, including marked thrombocytopenia and hemolytic anemia, which may pose significant health risks.^[10,11]^

Thrombocytopenia, defined by platelet counts falling below 100 × 10^9^/L, is a common hematological finding in SLE, occurring in approximately 10% to 40% of cases, and can present with a diverse spectrum of clinical forms, ranging from acute and severe to chronic or even asymptomatic cases.^[9,12]^ Notably, thrombocytopenia may serve as the initial presentation of SLE in approximately 5% to 16% of patients.^[13]^ Furthermore, SLE has been found to develop in around 2% of individuals initially diagnosed with primary immune thrombocytopenia.^[14]^

SLE primarily impacts women, particularly those aged 20 to 40. Pediatric-onset SLE is relatively uncommon, with only about 20% of all SLE cases occurring in patients under 16 years of age, and is associated with symptoms such as fever, fatigue, skin rashes, hematologic abnormalities, and mucosal ulcers.^[1,15]^ However, the disease can present in diverse ways, with some patients exhibiting atypical symptoms that may complicate diagnosis.^[3]^

In pediatric cases of SLE, the disease disproportionately affects females, but the female-to-male ratio is approximately 3:1—lower than the ratio observed in adult populations. This difference is thought to be due to the reduced influence of sex hormones in children, which plays a more significant role in the higher prevalence of SLE among adult females.^[16]^

Hematological abnormalities as the initial and sole manifestation of pediatric SLE are uncommon, with limited cases documented in the literature.^[3,6]^ These isolated blood-related symptoms can include hemolytic anemia, thrombocytopenia, and chronic leukopenia.^[3]^

To our review, there are very few documented cases of pediatric SLE presenting solely with hematological abnormalities. One such case, reported in 2015, described a 12-year-old boy who was initially diagnosed with SLE after presenting with isolated thrombocytopenia without any of the other typical clinical manifestations of SLE.^[6]^ Another case, reported in 2021, described an 8-year-old girl who was diagnosed with SLE after presenting with both thrombocytopenia and anemia as her first manifestations.^[3]^ A further case reported in 2022, described a 12-year-old girl initially diagnosed with ITP-causing anemia before a subsequent diagnosis of SLE was made.^[17]^

In our article, we present a case of atypical presentation of SLE in an 11-year-old male with isolated hematologic abnormalities as the initial manifestation. The patient’s presentation with thrombocytopenia, along with the family history of localized scleroderma, warranted further investigation into an autoimmune etiology. The elevated ANA, dsDNA, and complement abnormalities reinforced the diagnosis of pediatric-onset SLE.

The diagnosis of SLE in pediatric patients remains a significant challenge for clinicians. The disease presents with a wide range of heterogeneous clinical manifestations, often complicating its identification. Moreover, the potential impact of SLE on a child’s physical development and overall well-being underscores the critical need for early recognition and intervention.^[6]^ In our case, the ITP was the only manifestation that posed a diagnostic challenge. However, the patient’s positive family history of autoimmune diseases was a key factor that guided the decision to initiate a workup for SLE.

Pediatric SLE can present with atypical features, making early diagnosis challenging, particularly in adolescent females. A high index of suspicion is crucial, as timely identification and treatment are necessary to prevent adverse effects on growth and to halt disease progression.^[3,6]^ Our case highlights that isolated thrombocytopenia may serve as an early indicator of SLE. This underscores the importance of evaluating children with idiopathic thrombocytopenia for autoimmune markers such as ANA and dsDNA, as early detection can lead to more effective management and improved outcomes.

## 4. Conclusion

This case report presents an 11-year-old male who initially presented with isolated thrombocytopenia and pancytopenia, and was later diagnosed with cSLE. Despite lacking typical clinical features of SLE, the patient’s family history of autoimmune disease and persistent hematological abnormalities prompted further investigation. Positive autoimmune markers, including elevated ANA, anti-dsDNA, and complement abnormalities, confirmed the diagnosis. This underscores the importance of considering SLE in the differential diagnosis of patients presenting with isolated ITP.

## Acknowledgments

We would like to thank the patient’s family for cooperating in this study.

## Author contributions

**Supervision:** Fawzy M. Abunejma.

**Conceptualization:** Maaweya Jabareen.

**Validation:** Maaweya Jabareen.

**Writing—original draft:** Maaweya Jabareen.

**Writing—review & editing:** Maaweya Jabareen.

**Data curation:** Wasef Alhroub, Asrar Alhroub.

**Resources:** Wasef Alhroub, Asrar Alhroub.

**Investigation:** Bashar Qanaeer.

**Software:** Bashar Qanaeer, Adam Zaro.

**Methodology:** Abrar Zaro.
